# Mechanical Energy Recovery during Walking in Patients with Parkinson Disease

**DOI:** 10.1371/journal.pone.0156420

**Published:** 2016-06-03

**Authors:** Mariangela Dipaola, Esteban E. Pavan, Andrea Cattaneo, Giuseppe Frazzitta, Gianni Pezzoli, Paolo Cavallari, Carlo A. Frigo, Ioannis U. Isaias

**Affiliations:** 1 Department of Pathophysiology and Transplantation, Human Physiology Section, Università degli Studi di Milano, Milan, Italy; 2 Movement Biomechanics and Motor Control Lab, Dipartimento di Elettronica, Informazione e Bioingegneria, Politecnico di Milano, Milan, Italy; 3 Department of Neurology, University Hospital of Würzburg and Julius Maximilian University of Würzburg, Würzburg, Germany; 4 Moriggia-Pelascini Hospital, Gravedona ed Uniti, Italy; 5 Parkinson Institute, Pini-CTO (ex ICP) Milan, Italy; 6 Fondazione Europea di Ricerca Biomedica FERB Onlus, Milan, Italy; Hospital General Dr. Manuel Gea González, MEXICO

## Abstract

The mechanisms of mechanical energy recovery during gait have been thoroughly investigated in healthy subjects, but never described in patients with Parkinson disease (PD). The aim of this study was to investigate whether such mechanisms are preserved in PD patients despite an altered pattern of locomotion. We consecutively enrolled 23 PD patients (mean age 64±9 years) with bilateral symptoms (H&Y ≥II) if able to walk unassisted in medication-off condition (overnight suspension of all dopaminergic drugs). Ten healthy subjects (mean age 62±3 years) walked both at their ‘preferred’ and ‘slow’ speeds, to match the whole range of PD velocities. Kinematic data were recorded by means of an optoelectronic motion analyzer. For each stride we computed spatio-temporal parameters, time-course and range of motion (ROM) of hip, knee and ankle joint angles. We also measured kinetic (W_k_), potential (W_p_), total (W_totCM_) energy variations and the energy recovery index (ER). Along with PD progression, we found a significant correlation of W_totCM_ and W_p_ with knee ROM and in particular with knee extension in terminal stance phase. W_k_ and ER were instead mainly related to gait velocity. In PD subjects, the reduction of knee ROM significantly diminished both W_p_ and W_totCM_. Rehabilitation treatments should possibly integrate passive and active mobilization of knee to prevent a reduction of gait-related energetic components.

## Introduction

Gait disturbance is a relevant component of motor disability in subjects with Parkinson disease (PD) and a large amount of experimental work has been dedicated to investigate biomechanical abnormalities in these patients. While PD patients at an early disease stage can show exclusively a reduction of gait velocity and stride length [1–3], along with disease progression they usually exhibit shortened stride length, prolonged stance and double support phases [4,5] and reduced velocity [3–6]. Gait cadence might not be altered [7,8] or, in some cases, it appears to be increased as a possible adaptation to stride length reduction [6,8–10]. The range-of-motion (ROM) at lower limb joints is also usually reduced [8,11–15]. Very few studies, with unclear results, investigated energetic expenditure in PD patients. In particular, patients were investigated in unspecified meds-on state [16], or while walking on a treadmill [17], a condition which has been shown to alter the gait pattern with respect to over-ground walking [18,19]. In addition, PD patients were never compared to healthy subjects walking at similar velocities. Last but not least, the role of mechanical energy recovery was never taken into account in the analysis of energy expenditure along a stride cycle of PD patients.

In normal walking, the gravitational potential energy (E_p_) of the center of mass (CM) is at maximum level during mid stance, when the kinetic energy (E_k_) of the CM is minimum. From mid stance, the CM descends, and E_p_ is partially converted into E_k_; forward acceleration occurs and the body lands on the contralateral limb. After this foot-ground contact, the CM again moves upward (as long as the limb remains relatively straight extended) and decreases its forward velocity. As a consequence, E_p_ increases again and E_k_ decreases [19–21]. Energy variation corresponds to mechanical work, so that ΔE_k_ = W_k_ and ΔE_p_ = W_p_. In an ideal energy recovery mechanism, the work associated to changes of potential energy is exactly the same as the work associated to kinetic energy changes, but with different sign: W_p_ = -W_k_. That means that work produced to increase the potential energy can be obtained by reducing the kinetic energy, and can again be returned to increase the kinetic energy at the next step-to-step transition. Actually, the conversion between E_p_ and E_k_ does not occur completely, but it is about 70% during normal walking at preferred speed [21].

Several studies have separately indicated that the metabolic cost of walking is primarily allocated towards raising the CM throughout the gait cycle [22–24]. Therefore, the mechanism of exchanging E_k_ and E_p_ aims to reduce the metabolic cost of locomotion by lowering the muscular effort required to accelerate and decelerate the CM [25].

Aim of this study was to investigate changes in the mechanical energy recovery, and its correlations with spatio-temporal gait parameters, in a carefully selected cohort of PD patients at different disease stages.

## Materials and Methods

### Subjects

We consecutively enrolled 23 PD patients with bilateral symptoms (Hoehn and Yahr, HY stage ≥II) if able to walk unassisted in medication-off condition (overnight suspension of all dopaminergic drugs). All patients had stable dopaminergic treatment for at least six months and no levodopa-related motor fluctuations (e.g., dyskinesia). Ten age-matched healthy subjects (HC, mean age 62±3 years) also took part in the study. The diagnosis of PD was made according to the UK Brain Bank criteria and patients were evaluated with the Unified Parkinson Disease Rating Scale motor part (UPDRS-III). All PD patients improved (>20% at UPDRS-III score) after intake of 150–200 mg of L-Dopa (acute challenge test), thus further supporting the clinical diagnosis of idiopathic PD. Patients were not suffering from freezing of gait and did not show any freezing episodes during the acquisitions. No patients showed any atypical features of parkinsonism. Patients with cognitive decline (Mini-Mental State Examination <27) or any other signs of neurological or psychiatric disease other than PD were excluded. All patients did not suffer from any other disease than PD nor underwent any major surgery (e.g. orthopedic surgery). Patients were divided into two groups according to the HY stage: mild group (PD_M_: HY stage II), and severely affected group (PD_S_: HY stage III or IV). The local institutional review board (Section of Human Physiology, Department of Pathophysiology and Transplantation, University of Milan) approved the study and the consent procedure. All participants signed a written informed consent. All efforts were made to protect patient privacy and anonymity.

### Experimental Setup and Protocol

Kinematic data were recorded using an optoelectronic system (SMART-E, BTS Bioengineering, Italy), consisting of six video cameras (sampling rate: 60 Hz; calibrated volume 4x2x1.5m). The position of the subjects’ main body segments was determined by means of 29 retro-reflective markers (diameter: 15 mm) according to a published protocol [1]. During the static calibration trial, eight additional “technical” markers were attached on the following bony landmarks, on both sides of the body: greater trochanter, medial femoral condyle, medial malleolus, and first metatarsal head. The position of these points, not visible to the cameras during gait, was computed offline by means of technical reference systems, assuming their relative position in relation to local reference frames was fixed. Anthropometric parameters of each subject were computed from the markers’ positions recorded during the calibration trial, and used for the estimation of internal joint centers, thus enabling calculation of lower limb kinematics. Subjects were asked to walk barefoot along a straight trajectory about 11.5 m long. All subjects started to walk from at least two strides behind the calibrated volume, without any starting command. Trials were repeated three to five times, according to patients' capabilities. All PD patients were evaluated after overnight suspension of all dopaminergic drugs (meds-off).

HC did two sets of eight walking trials at ‘preferred’ (HC_N_) and ‘slow’ (HC_S_) speeds, in random order, following verbal instructions in the absence of external feedback. The consistency of the two datasets was verified on the basis of the actual measured speeds.

### Data Analysis and energy calculation

We used ad-hoc algorithms to compute the CM trajectory all along the gait cycle and to measure spatio-temporal gait parameters (i.e. walking speed, stride length and period, stance phase and double support phase duration), time courses of hip, knee and ankle joints angles during the stride cycle and their ROM.

For each subject, spatial parameters were normalized as a percentage of the body height (BH). Temporal parameters and all curves representing the time-course of kinematic variables were time normalized as a percentage of the stride duration (defined from heel contact of one foot to next heel contact of the same foot).

Subsequently, the mean values and the standard deviation (SD) for corresponding normalized time intervals were calculated for each variable of each subject. ROM values were computed as the difference between the maximum and the minimum values reached by each joint angle, within the stride.

The whole body center of mass (CM) was computed by estimating the displacement of the center of mass of each body segment (CM_j_), and then implementing the general formula:

$$Y_{CM}=\frac{\sum_{j}y_{j}\;m_{j}}{M}$$ (1)

where, Y_CM_ is the generic coordinate of CM; y_j_ is the generic coordinate of center of mass of each anatomical segment (j), m_j_ is the mass of each body segment (j) and M is the mass of the whole body. The position of CM_j_ within each anatomical segment, as well as the mass of each body segment were obtained from the anthropometric tables and regression equations provided by Zatsiorsky and Seluyanov [26].

The kinetic energy associated to CM displacements was computed as follows:

$$E_{k,CM}=\frac{1}{2}\;M\;({v_{x}}^{2}+{v_{y}}^{2}+{v_{z}}^{2})$$ (2)

where M is the whole body mass, v_x_, v_y_, v_z_ are the three components of the velocity of CM.

The potential energy associated to CM was calculated as:

$$E_{p,CM}=Mgh$$ (3)

where g is the gravitational acceleration (m/s^2^) and h is the vertical distance of CM from the ground.

The total energy associated to CM was computed as function of time (t) as:

$$E_{tot,CM}(t)=E_{k,CM}(t)+E_{p,CM}\;(t)$$ (4)

Over the stride period, the positive variations (difference between maximum and minimum) of respectively E_p,CM_, E_k,CM_, and E_tot,CM_ were identified and calculated. They were named respectively W_p_, W_k_, and W_totCM_. Then the energy recovery index (ER) was computed according to Cavagna et al. [21]:

$$ER=\frac{(W_{p}+W_{k})-W_{totCM}}{\left(W_{p}+W_{k}\right)}\times 100$$ (5)

All energetic parameters were computed for each stride collected from our subjects and averaged over the strides (left and right pooled together) for each gait velocity command and for each group of subjects. Energetic parameters were divided by the mass (M) of the subject.

### Statistics

Statistical analysis was performed using JMP statistical package (version 12.0, SAS Institute, Inc., Cary, NC, USA). ROMs of left and right hemibodies were compared by means of matched pairs analysis. Differences between PD and HC groups were analyzed by means of Kruskal-Wallis and Steel-Dwass tests. To look for measurements that were predictive of energetic parameters, we used the Spearman correlation coefficient. Variables were then included in stepwise multiple linear regression. Strength of the correlations was defined according to the absolute value of *ρ* as “very weak” (.00-.19), “weak” (.20-.39), “moderate” (.40-.59), “strong” (.60-.79) and “very strong” (.80–1.0). A p<0.05 was considered to be statistically significant.

## Results

Demographic and clinical data are listed in Table 1. As expected, PD_S_ showed higher scores at UPDRS-III in meds-off and higher L-Dopa Equivalent Daily Dose (LEDD) [27] compared to PD_M_ (p<0.05, in both cases). No significant differences were found for age and body mass index among PD sub-groups and HC. No difference was also found when comparing ROMs of the right and left hemibodies, both for HC and PD, and data were then pooled together.

**Table 1:** Demographic and clinical data.

	PD_M_	PD_S_	HC
**N. (male/female)**	10 (8/2)	13 (7/6)	10 (8/2)
**Age (years)**	62 ± 9	65 ± 8	62 ± 3
**Weight (kg)**	80.2 ± 14.6	65.0 ± 13.7	80.8 ± 9.5
**Height (m)**	1.7 ± 0.1	1.6 ± 0.1	1.7 ± 0.1
**BMI**	27.7 ± 5.2	24.5 ± 4.7	26.9 ± 3.1
**Disease duration (years)**	5 ± 2	12 ± 3	
**UPDRS-III**	20 ± 9	28 ± 9	
**L-Dopa daily dose**	325.0 ± 143.6	557.1 ± 225.0	
**LEDD**	443.3 ± 142.4	690.4 ± 205.6	

LEDD = L-Dopa Equivalent Daily Dose; UPDRS-III = Unified Parkinson’s Disease Rating Scale motor part (III) in meds-off state; BMI = Body mass index. Disease duration was from motor symptoms onset. Values are means and standard deviation.

**Fig 1:**
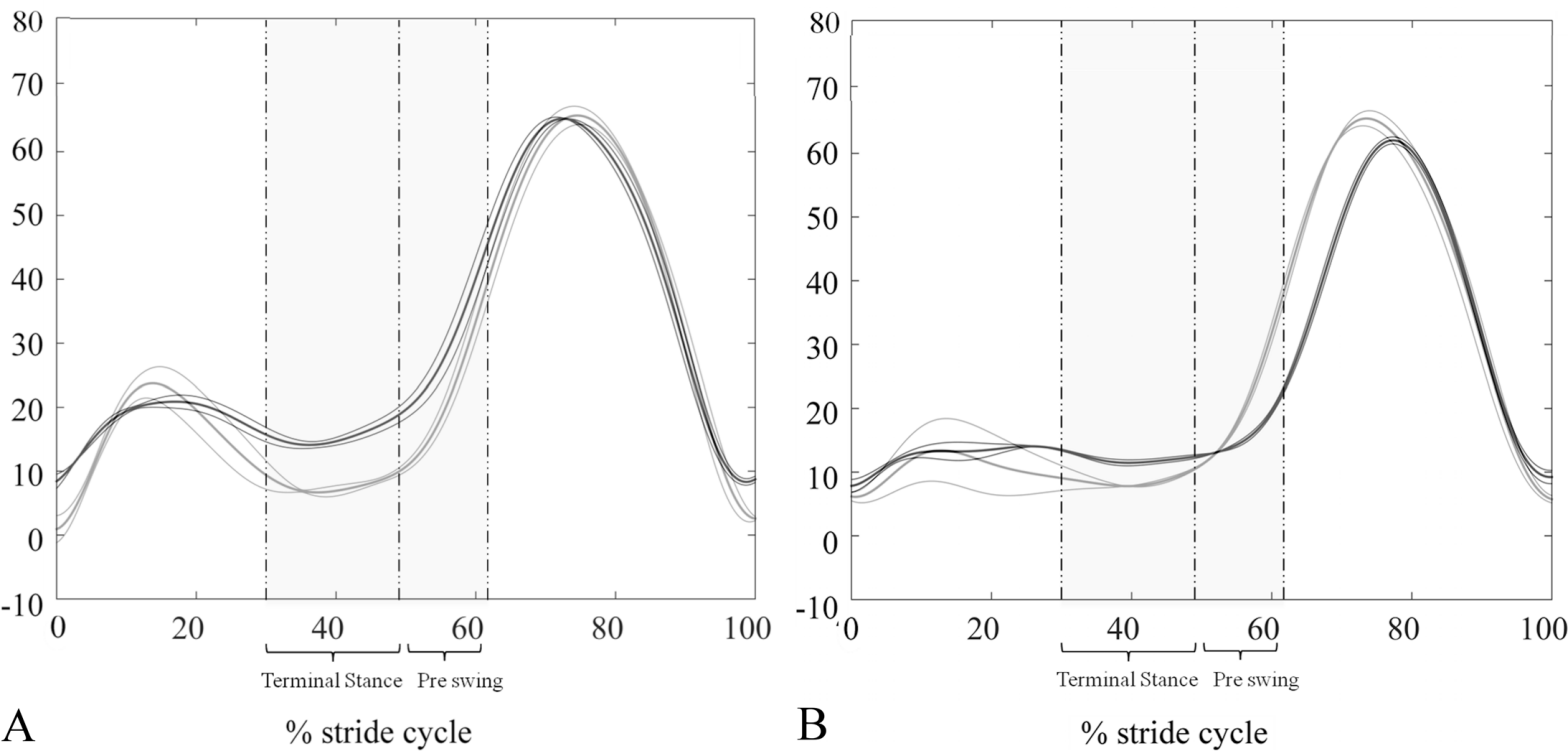
Time courses of knee flexion/extension angles. (A) Comparison between one representative PD_M_ (black lines) and one HC_N_ (grey lines). (B) Comparison between one representative PD_S_ (black lines) and one HC_S_ (grey lines). Thick and thin lines refer to the average time courses ±SD of different trials, respectively. The intervals of maximum knee extension, reached during the stance phase, are highlighted in grey.

Spatio-temporal, kinematic and energetic parameters are listed in Table 2. Average gait velocity of HC_N_ matched the homologous data of PD_M_, and the same hold true concerning HC_S_ and PD_S_. Of relevance, PD_S_ showed a significant reduction of stride length and knee ROM when compared to HC_S_, which was due to a more flexed knee in the stance phase (Fig 1). The average knee joint angles measured during terminal stance were: 12.74±5.32° and 6.22±4.62° for PD_M_ and HC_N_ (p<0.05), and 13.52±8.24° and 5.19±4.42° for PD_S_ and HC_S_ (p<0.05). Lastly, in PD_S_ hip and ankle ROMs were reduced in comparison to PD_M_ and both negatively correlated with UPDRS-III scores (hip ROM: *ρ* = -0.56, p<0.05 and ankle ROM: *ρ* = -0.54, p<0.05).

**Table 2:** Energetic, spatio-temporal and kinematic parameters.

Parameters	HC_N_	HC_S_	PD_M_	PD_S_
**Stride Velocity (%BH/s)**	67.4±6.0 ^1*^	43.3±8.1 ^1^	64.2±5.6 ^2*^	41.1±8.7 ^2^
**Stride Period (s)**	1.1±0.1 ^1*^	1.4±0.2 ^1^	1.1±0.1 ^2^	1.2±0.1 ^2^
**Stride Length (%BH)**	71.6±5.2 ^1^	59.3±5.9 ^1, 3^	68.8±5.8 ^2*^	49.0±6.6 ^2, 3^
**% Stance Phase**	61.8±2.1 ^1*^	66.5±2.8 ^1^	61.1±1.8 ^2^	64.7±3.4 ^2^
**% Double Support Phase**	12.1±2.2 ^1^	16.4±3.2 ^1^	11.4±1.9 ^2*^	16.0±2.5 ^2^
**Hip ROM (°)**	40.9±2.9 ^1*^	35.8±2.1 ^1^	38.0±6.6 ^2*^	31.0±8.2 ^2^
**Knee ROM (°)**	56.0±4.7	53.8±3.8 ^3^	49.6±8.3	42.0±9.3 ^3^
**Ankle ROM (°)**	24.5±4.3	22.1±5.1	26.8±4.7 ^2*^	20.4±6.2 ^2^
**ER index (%)**	65.4±5.7 ^1^	49.1±10.2 ^1^	68.2±4.3 ^2^	52.5±12.13 ^2^
**W**_**totCM**_ **(J/kg)**	0.36±0.08	0.37±0.05 ^3*^	0.34±0.05 ^2*^	0.24±0.04 ^2, 3^
**W**_**p**_ **(J/kg)**	0.57±0.14	0.47±0.09 ^3^	0.57±0.06 ^2*^	0.33±0.09 ^2, 3^
**W**_**k**_ **(J/kg)**	0.51±0.08 ^1*^	0.32±0.07 ^1, 3^	0.5±0.09 ^2*^	0.21±0.06 ^2, 3^

Superscript numbers indicate statistically significant differences (p<0.05 or p<0.01 when * is present) between HC_N_ and HC_S_ (1), PD_S_ and PD_M_ (2), HC_S_ and PD_S_ (3). We did not find any statistical difference between HC_N_ and PD_M_. Values are means and standard deviation. See text for statistical analysis.

**Fig 2:**
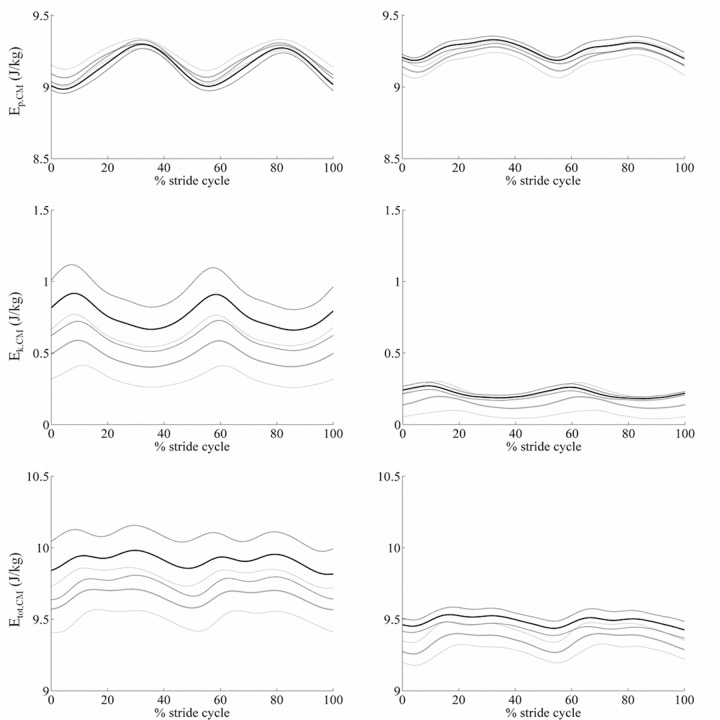
Energy components. Left column: PD_M_ and HC_N_. Right column: PD_S_ and HC_S_. Black lines refer to one representative PD and grey lines to one HC. Thick and thin lines refer to the average time courses ±SD of different trials, respectively.

In Table 2 we listed all energetic measurements. Fig 2 shows time courses of kinetic, potential and total energy associated to CM during the stride cycle. As expected, we found low values of ER and W_k_ in slow walking subjects (i.e. PD_S_ and HC_S_) being both measurements strictly related to gait velocity. Indeed, ER and W_k_ correlated with stride velocity in HC (*ρ* = 0.82, p<0.0001 and *ρ* = 0.88, p<0.001, respectively) and in PD patients (*ρ* = 0.69, p<0.001 and *ρ* = 0.91, p<0.0001, respectively).

PD_S_ showed lower W_p_ values in comparison with PD_M_ and, more interestingly, with HC_S_, although walking at comparable velocities. W_p_ positively correlated with hip ROM in HC (*ρ* = 0.73, p<0.01) while with knee ROM in PD (*ρ* = 0.68, p<0.01). W_k_ values were lower in PD_S_ than HC_S_. Besides the aforementioned correlation with stride velocity, in PD patients W_k_ also correlated with hip ROM (*ρ* = 0.79, p<0.001). W_totCM_ matched closely with W_p_ findings. All mean data per subject were listed in S1 Table.

## Discussion

The main finding of our study was a reduction of W_totCM_ and W_p_ along with PD progression. These changes were greatly dependent on knee ROM reduction and in particular on knee extension in the terminal stance phase of the stride. W_k_ was also reduced in advanced PD patients primarily due to low gait velocities, but also due to a reduction of hip ROM, possibly reflecting a greater rigidity and stopped posture in more advanced stages of the disease (i.e. PD_S_).

The direct correlation of all energetic parameters with gait velocity [28,29], as seen also when comparing cohorts matched for gait velocity (Table 2), is reasonable if we consider that W_k_ is the variation of kinetic energy along the stride, and thus it depends on the square of velocity, and W_p_ is the variation of potential energy, which relies upon the vertical excursion of CM. Of note, the minimum height of CM excursion is reached during the double support phase, and depends on step length, which in turn is related to hip joint excursion and on gait velocity. The maximum height of CM excursion depends instead on how much the knee is extended in the mid stance phase.

In PD_S_, we found two main conditions to justify a reduction of all energetic components. In particular, (i) a reduced gait velocity, mainly as a result of short stride length (stride time was even shorter than in HC_S_) and (ii) a reduced hip and knee ROM, the latter resulting mainly from a lack of full extension in the terminal stance. Of note, in normal subjects the rate of knee extension in terminal stance should be approximately half that of flexion during limb loading [28]. In PD_S_, a deeply flexed knee during stance resulted in a reduced rising and a more flat path of the CM [25] which in turns reduced the amount of stored gravitational E_p_ [25,29,30]. We speculate that such an increased knee flexion could be related to an altered activity of plantar flexors muscles [9], which normally play a role to accelerate the knee into extension [12]. Indeed, in terminal stance the triceps surae muscle increases its activity and contracts vigorously as an ankle stabilizer [28]. The lack of EMG recording prevents us from confirming this hypothesis, but a reduction in amplitude of gastrocnemius activity was previously described in PD patients [12,13].

Of relevance, the finding that W_k_, W_p_, and W_totCM_ were reduced in PD_S_ in comparison to both patients walking faster (i.e. PD_M_) and control subjects walking at a similar velocity (i.e. HC_S_) suggests that such a reduction is mainly related to different kinematic patterns (such as altered ROMs) rather than to gait velocity *per se*.

At this point, it was quite unexpected that, despite a considerable reduction of all energetic parameters, the ER index itself was not reduced in PD_S_ when compared to HC_S_ subjects. This may suggest that the basic energy recovery mechanism, as adopted by normal subjects, which is an efficient way to reduce the energetic cost of walking, is still exploited in PD patients, also at advanced disease stages. Still, in PD_S_ the ER was relatively low when compared to subjects walking faster (both PD_M_ and HC_N_). Therefore, it can be argued that any intervention aimed at increasing gait velocity would be beneficial from the energetic point of view. However, it must be considered that lower limb can only roughly be approximated to an inverted pendulum for which perfect out-of-phase kinetic and potential energy variations occur. Actually, among the many factors that can reduce energy cost during walking, the knee flexion-extension at load acceptance, the ankle plantarflexion-dorsiflexion at early stance phase and pelvis tilt in the frontal plane in mid stance are the most important [31,32]. They all must be regarded with great attention if walking efficiency, as manifested by the ER index, is to be preserved.

A limitation of our study is the relative low number of patients recruited. This relied mainly upon our inclusion criteria. In particular, only few PD patients at HY stage III or IV were willing and able to suspend overnight all dopaminergic medications and even fewer were able to complete unassisted all walking trials in meds-off state. In this study, we recruited solely subjects with PD at HY stage II or higher as patients with mild motor symptoms can show normal ROM of hip, knee and ankle joints during linear walking at preferred speed ([1] and Table 2).

At last, our findings could help developing a tailored rehabilitation treatment of gait in PD subjects. Indeed, PD patients could benefit from passive and active mobilization of the knee to possibly normalize knee extension and consequently improve W_totCM_ and W_p_. Knee extension should be also monitored and possibly reinforced during treadmill training, which has been proved useful in the rehabilitation of gait disorders in PD, in particular at an early stage of the disease [33–35].

## Supporting Information

S1 Table Demographic, clinical, energetic, spatio-temporal and kinematic mean data of each subject. (PDF)
