# Supplementary material for: Mechanical Energy Recovery during Walking in Patients with Parkinson Disease
Source: PLoS One. 2016 Jun 3;11(6):e0156420. doi: 10.1371/journal.pone.0156420 (PMC4892681; doi:10.1371/journal.pone.0156420)
Supplement: S1 Table — (PDF) [file pone.0156420.s001.pdf]

Groups	Subject code	Gender	Height [m]	Body Weight [kg]	IBM	UPDRS-III	ER [%]	WtotCM [J/kg]
**HC-N**	COCM	F	1.54	63.66	27.02		62.35	0.3
**HC-N**	COAG	F	1.7	91.46	31.57		62.18	0.29
**HC-N**	COPB	M	1.83	81.03	24.18		53.07	0.53
**HC-N**	COFC	M	1.76	95.52	30.96		67.16	0.41
**HC-N**	COGM	M	1.76	70.57	22.84		69.93	0.27
**HC-N**	COCP	M	1.73	76.03	25.5		72.81	0.39
**HC-N**	COML	M	1.83	77.29	23.17		66	0.36
**HC-N**	COPM	M	1.73	86.36	28.96		68.63	0.35
**HC-N**	COPV	M	1.7	84.08	29.13		66.76	0.38
**HC-S**	COCM	F	1.54	63.66	27.02		35.98	0.36
**HC-S**	COAG	F	1.7	91.46	31.57		46.87	0.29
**HC-S**	COPB	M	1.83	81.03	24.18		40.6	0.45
**HC-S**	COAA	M	1.79	82.8	25.76		62.42	0.36
**HC-S**	COFC	M	1.76	95.52	30.96		41.72	0.35
**HC-S**	COGM	M	1.76	70.57	22.84		48.9	0.4
**HC-S**	COCP	M	1.73	76.03	25.5		64.75	0.36
**HC-S**	COML	M	1.83	77.29	23.17		60.77	0.37
**HC-S**	COPM	M	1.73	86.36	28.96		40.64	0.44
**HC-S**	COPV	M	1.7	84.08	29.13		48.43	0.4
**PD-M**	PD01	M	1.84	98.13	28.93	28	68.5	0.35
**PD-M**	PD02	M	1.74	69.88	23.15	21	68.56	0.37
**PD-M**	PD04	M	1.8	83.11	25.61	17	64.41	0.39
**PD-M**	PD07	F	1.61	100.72	38.85	25	77.28	0.22
**PD-M**	PD08	M	1.62	70.67	26.81	15	68.6	0.39
**PD-M**	PD09	M	1.63	70.47	26.57	34	62.81	0.32
**PD-M**	PD10	M	1.79	97.46	30.37	20	68.26	0.34
**PD-M**	PD12	F	1.7	56.51	19.67	7	67.6	0.35
**PD-M**	PD13	M	1.57	77.45	31.52	8	72.32	0.31
**PD-M**	PD14	M	1.72	77.45	26.27	23	63.53	0.35
**PD-S**	PD03	F	1.5	51.42	22.94	26	67.64	0.21
**PD-S**	PD15	M	1.72	62.3	21.1	21	32.08	0.22
**PD-S**	PD16	F	1.54	49.03	20.74	18	40.4	0.22
**PD-S**	PD17	M	1.63	61.15	22.95	27	45.3	0.25
**PD-S**	PD11	F	1.59	46.3	18.22	21	58.25	0.21
**PD-S**	PD18	M	1.84	66.53	19.75	31	49.25	0.31
**PD-S**	PD19	M	1.76	77.44	25.02	34	71.85	0.22
**PD-S**	PD20	M	1.66	77.91	28.27	48	35.96	0.3
**PD-S**	PD05	M	1.7	84.8	29.36	42	52.25	0.21
**PD-S**	PD06	F	1.56	74.8	30.56	30	53	0.24
**PD-S**	PD21	F	1.57	67.21	27.37	22	52.73	0.22
**PD-S**	PD22	M	1.58	80.55	32.37	21	56.76	0.31
**PD-S**	PD23	F	1.53	45.56	19.35	29	67.05	0.19

Wp [J/kg]	Wk [J/kg]	Stride Velocity [%BH/s]	Stride Period [s]	Stride Length [%BH]	Stance [%stride]
0.35	0.47	81.34	0.95	76.22	59.6
0.39	0.39	67.14	0.91	60.51	60.16
0.59	0.51	61.33	1.16	71.57	62.15
0.7	0.54	68.31	1.05	71.73	61.9
0.5	0.41	62.79	1.11	67.71	61.65
0.79	0.64	69.25	1.13	77.6	61.84
0.57	0.53	67.43	1.08	71.66	61.83
0.62	0.52	61.63	1.23	75.86	66.77
0.6	0.57	67.6	1.06	71.81	60.58
0.36	0.24	33.85	1.9	57.98	68.56
0.34	0.25	43.65	1.21	51.11	63.42
0.45	0.32	36.97	1.49	54.63	68.09
0.58	0.4	49.01	1.37	66.38	65.3
0.43	0.21	31.88	1.69	50.67	70.04
0.47	0.31	54.62	1.15	63.15	64.34
0.54	0.45	55.8	1.19	66.6	62.24
0.6	0.36	42.79	1.45	61.32	68.01
0.46	0.28	40.09	1.6	63.7	70.09
0.46	0.33	44.48	1.29	57.68	65.21
0.59	0.51	61.23	1.03	62.44	62.85
0.58	0.6	71.12	1.02	71.95	59.17
0.62	0.5	64.37	1.12	71.61	59.29
0.56	0.41	63.46	1.16	73.28	61.57
0.66	0.54	66.7	1.12	73.3	60.83
0.51	0.4	68.16	1.05	71.22	62.03
0.58	0.5	58.23	1	58.06	61.61
0.45	0.62	73.19	1.03	75.51	59.72
0.54	0.58	60.75	1.13	68.26	59.6
0.59	0.37	55.3	1.14	62.32	64.67
0.4	0.26	45.3	1.2	52.77	63.4
0.18	0.17	36.73	1.25	46.56	62.94
0.21	0.15	32.51	1.5	48.78	69.42
0.35	0.11	31.97	1.37	42.89	66.34
0.33	0.19	33.36	1.39	42.65	69.55
0.36	0.27	43.21	1.34	58.31	56.79
0.47	0.31	47.16	1.18	52.86	64.09
0.34	0.13	35.03	1.25	46.86	64.9
0.27	0.17	29.32	1.2	34.13	67.7
0.3	0.25	47.2	1.08	50.99	63.59
0.25	0.23	48.14	1.08	50.42	63.98
0.49	0.23	44.8	1.18	50.71	66.68
0.35	0.24	59.29	1.01	58.66	62

Double [%stride]	Hip Angle ROM Left (deg)	Hip Angle ROM Right (deg)	Knee Angle ROM Left (deg)
10	41.84	39.19	56.16
9.44	41.52	41.9	51.76
12	42.18	42.24	52.01
12.7	44.91	40.09	56.37
12.79	35.51	36.79	53.76
12.22	42.88	42.62	58.97
12.21	42.88	42.62	58.97
17.02	45.19	41.28	67.16
10.75	36.36	36.62	57.43
18.87	33.83	37.46	52.9
13.32	38.99	32.7	54.85
18.65	34.65	35.75	50.92
15.36	36.41	35.58	59.14
19.99	33.65	33.86	47.76
13.72	34.89	35.44	53.9
11.19	36.56	39.6	53.56
17.97	34.22	38.72	55.04
20.38	39.01	36.48	61.92
14.82	33.93	33.93	56.41
12.3	36.54	40.12	59.96
10.15	36.49	39.8	50.65
10.87	42.51	41.84	59.84
12.26	28.96	29.12	34.64
10.54	52.24	42.16	
10.7	37.18	34.26	51.04
11.82	29.72	26.11	39.27
10.39	41.65	41.55	52.49
8.87	46.32	45.72	44.61
15.77	35.96	32.68	54.06
13.36	39.8	41.2	36.84
17.28	31.43	30.3	50.76
16.48	40.18	39.79	42.89
17.98	25.87	29.5	35.29
19.08	31.43	30.3	50.57
11.59	39.01	39.63	55.22
14.62	35.53	35.92	46.68
17	27.16	18.33	33.75
19.34	35.53	35.92	46.68
15.1	12.28	14.24	22.22
15.82	38.16	25	48.89
18.29	31.94	31.47	56.35
12.04	27.16	18.33	33.75

Knee Angle ROM Right (deg)	Ankle Angle ROM Left (deg)	Ankle Angle ROM Right (deg)
	19.39	26.62
	25.11	28.07
48.14	26.79	21.39
59.79	19.85	21.63
47.4	22.43	20.25
56.49	25.86	25.11
56.49	25.86	25.11
57.19	37.89	26.44
57.72	21.38	22.16
55.59	14.78	19.4
51.15	25.95	20.84
46.02	24.91	19.73
51.38	27.22	25.5
53.32	12.83	16.58
48.52	22.75	20.91
57.65	21.5	22.96
56.41	21.49	18.7
55.8	36.68	27.29
53.7	21.3	21.27
	23.49	30.1
50.19	26.87	23.62
55.27	23.53	31.49
35.25	28.8	26.61
55.45	25.59	26.61
53.75	21.33	22.4
35.16	22.08	18.99
52.69	34.07	31.83
53.93	22.15	36.62
55.26	31.66	28.9
42.81	15.82	15.22
44.06	18.07	17.32
35.35	25.17	31.21
31.81	17.31	18.21
46.96	21.07	16.54
54.13	18.37	18.54
42.99	18.61	25.67
36.37	31.2	21.14
42.99	18.61	25.67
28.29	7.52	7.7
32.65	14.67	25.21
57.91	23.74	24.7
36.37	31.2	21.14
